# Advancing mental health in the Americas: challenges and opportunities

**DOI:** 10.1590/0102-311XEN162325

**Published:** 2025-10-03

**Authors:** Alessandra Trianni, Amy Tausch, Renato Oliveira e Souza

**Affiliations:** 1 Pan American Health Organization, Washington DC, U.S.A.

Mental health is considered an urgent priority in the Americas, in part, due to recognition of the widespread impacts of the COVID-19 pandemic. Yet, despite the significant disability and premature mortality associated with mental disorders, countries in this region designate only a small fraction of their health budgets to such conditions and the majority of the population has limited access to community-based services. Data, although scarce, indicate that populations living in situations of vulnerability face greater barriers to mental health services. Serpeloni et al. ^1^, Queiroz et al. ^2^, and Orth et al. ^3^ point out shortcomings in mental health care for populations in vulnerable situations, specifically those affected by conflict and violence and incarcerated individuals, underscoring the need for greater investments, cross-sectoral collaboration, and research in mental health in order to expand access to services and reach those with the greatest need. In this context, recent policy developments on the regional level offer an important window of opportunity.

The disease burden attributable to mental health conditions in the Americas is high and rising. Mental, neurological, and substance use (MNS) disorders and suicide account for over one-third of years lived with disability (YLDs) and nearly a fifth of all disability-adjusted life years (DALYs) in the region ^4^. Between 2000 and 2019, the mortality rate from MNS disorders rose by 89% and the rate of DALYs rose by 10% ^5^. Mental health conditions are also a risk factor for suicide, which alone claimed 100,000 lives in the region in 2021. Concerningly, the suicide rate increased by 17% between 2000 and 2021 in the Americas, which was the only one of the regions delineated by the World Health Organization (WHO) to experience a rise in this period ^6^. The COVID-19 pandemic further exacerbated the mental health situation by amplifying known risk factors for mental health conditions, such as social isolation, unemployment, poverty, and violence, and disrupting already fragile mental healthcare systems and services. In 2020, major depressive disorders and anxiety disorders increased by an estimated 35% and 32%, respectively, in Latin America and the Caribbean due to the pandemic ^7^.

Despite this burden, mental health systems in the region remain chronically underfunded. A median of only 3% of national health budgets is allocated to mental health, nearly half of which goes to long-stay psychiatric institutions, which are often associated with poor outcomes and human rights violations ^8^. In many countries, the availability and accessibility of community-based mental health services are severely limited, resulting in a large treatment gap, as only an estimated 18% of individuals experiencing psychosis receive treatment ^8^. It is likely that the gap is larger in certain population groups, but the lack of disaggregated data makes it difficult to calculate this aspect.

Recognizing the mounting mental health crisis in the region, the member States of the Pan American Health Organization (PAHO) adopted the Policy for Improving Mental Health in 2023, which explicitly framed mental health as essential to sustainable development and recovery from the COVID-19 pandemic ^9^. The following year, PAHO adopted the Strategy for Improving Mental Health and Suicide Prevention in the Region of the Americas ^10^, with the aim of “*improving mental health and suicide prevention, using an equity- and human rights-based approach, in order to advance health and development in the Region*”. A key driver behind the strategy was the final recommendations of the PAHO High-Level Commission on Mental Health and COVID-19 outlined in the report *A New Agenda for Mental Health in the Americas* ^11^.

Regional momentum for mental health has recently reached unprecedented levels through the inclusion of the issue on the hemispheric agenda of the Organization of American States (OAS). In June 2025, OAS member states unanimously approved a historic resolution ^12^ to address the mental health crisis in the Americas, which establishes a regional mental health partnership to strengthen collaboration, expand access, and promote the implementation of community-based mental health initiatives. The resolution also calls for the formation of an inter-American working group to develop an action plan aligned with the regional mental health strategy as well as the creation of a regional mental health fund to bolster priority mental health initiatives. The passage of this resolution constitutes an important step towards placing mental health on the broader political agenda of the Americas, where mental health has historically been considered an issue solely of the health sector.

While political commitment on the regional level has grown, it is harder to measure how this progress translates to the national level. Countries in the region have undoubtedly made crucial advances in strengthening their mental health systems in recent years, especially through the development and updating of mental health legislation and policies that are better aligned with international human rights standards and in transitioning towards community-based approaches to mental health care. A central pillar of this work has been the integration of mental health services into primary care using the WHO Mental Health Gap Action Program (mhGAP) ^13^. Importantly, nearly all countries in the region have carried out capacity building in mhGAP. However, as Queiroz et al. ^2^ point out, there are numerous challenges to implementing this program in different settings and few studies have assessed its impact in terms of improved access to mental health services on the primary care level.

Indeed, all three studies cited above point to the need for expanded mental health research to provide information that can serve as the basis for effective policies that target where the need is greatest. Implementation science, in particular, is essential to assessing, adapting, and scaling up evidence-based practices and tools for mental health in diverse local settings across the region. Increased investment in mental health - both financial and in the form of workforce development - in addition to collaboration across sectors, including but not limited to emergency preparedness and response, violence prevention, and justice, are needed to reduce the large mental health treatment gap in the Americas and ensure that no one is left behind.

The Region of the Americas stands at a critical juncture in its efforts to transform mental health into a public health and development priority. While the burden of mental health conditions continues to rise, recent years have seen unprecedented political momentum in terms of addressing this issue. Looking ahead, the challenge becomes translating this commitment into effective change on the country level, which is a task that will require sustained political will, investment, and multisectoral action as well as data, evidence, and research to guide it.
