# Supplementary material for: Advancing mental health in the Americas: challenges and opportunities
Source: Cad Saude Publica. 2025 Oct 3;41(9):e00162325. doi: 10.1590/0102-311XEN162325 (PMC12494381; doi:10.1590/0102-311XEN162325)
Supplement: Translation

# Translation: Avançando a saúde mental nas Américas: desafios e oportunidades

**DOI:** 10.1590/0102-311XPT162325

**Authors:** Alessandra Trianni, Amy Tausch, Renato Oliveira e Souza

**Affiliations:** 1 Pan American Health Organization, Washington DC, U.S.A.

A saúde mental é considerada prioridade urgente nas Américas, devido, em parte, ao reconhecimento dos impactos generalizados da pandemia de COVID-19. No entanto, apesar da significativa incapacidade e mortalidade prematura associadas aos distúrbios mentais, os países desta região destinam apenas uma pequena fração de seus orçamentos de saúde para tais condições e a maioria da população tem acesso limitado a serviços comunitários de saúde mental. Informações, embora escassas, indicam que as populações que vivem em condições de vulnerabilidade enfrentam maiores barreiras aos serviços de saúde mental. Serpeloni et al. ^1^, Queiroz et al. ^2^ e Orth et al. ^3^ apontam as deficiências na assistência à saúde mental para as populações em situações de vulnerabilidade, especificamente aquelas atingidas por conflitos e pela violência, bem como indivíduos encarcerados, ressaltando a necessidade de maiores investimentos, colaborações intersetoriais e pesquisas em saúde mental para ampliar o acesso aos serviços e alcançar aqueles com maiores necessidades. Avanços recentes relacionados às políticas em nível regional oferecem importante janela de oportunidade.

A carga de doenças atribuíveis aos distúrbios mentais nas Américas é alta e crescente. Transtornos mentais, neurológicos e por uso de substâncias são responsáveis por mais de um terço dos anos vividos com incapacidade e quase um quinto de todos os anos de vida ajustados por incapacidade na região ^4^. Entre 2000 e 2019, a taxa de mortalidade por tais transtornos aumentou em 89% e a taxa de anos vividos com incapacidade aumentou em 10% ^5^. Os distúrbios mentais também se configuram como fatores de risco para o suicídio, que por si só levou 100 mil vidas na região no ano 2021. De modo desconcertante, a taxa de suicídio aumentou em 17% entre 2000 e 2021 nas Américas, que foi a única das regiões delineadas pela Organização Mundial da Saúde (OMS) a experimentar um aumento neste período ^6^. A pandemia de COVID-19 agravou ainda mais a situação da saúde mental, ampliando os fatores de risco conhecidos para os distúrbios mentais, como isolamento social, desemprego, pobreza e violência, e interrompendo os já frágeis sistemas e serviços de saúde mental. No ano 2020, os transtornos depressivos maiores e os transtornos de ansiedade aumentaram em aproximadamente 35% e 32%, respectivamente, na América Latina e no Caribe devido à pandemia ^7^.

Apesar da carga desses distúrbios, os sistemas de saúde mental na região permanecem cronicamente subfinanciados. Uma mediana de apenas 3% dos orçamentos nacionais de saúde é alocada à saúde mental, quase metade da qual vai para as instituições psiquiátricas de longa permanência, frequentemente associadas a resultados insatisfatórios e violações de direitos humanos ^8^. Em muitos países, a disponibilidade e a acessibilidade de serviços de saúde mental comunitários são severamente limitadas, resultando em grande lacuna no tratamento. De fato, estima-se que apenas 18% das pessoas em situações de psicose recebam tratamento ^8^. É provável que a lacuna seja maior em certos grupos populacionais, mas a falta de dados desagregados dificulta o cálculo desse aspecto.

Reconhecendo a crescente crise de saúde mental na região, os Estados-Membros da Organização Pan-Americana da Saúde (OPAS) adotaram a Política para a Melhoria da Saúde Mental em 2023, que colocou explicitamente a saúde mental como essencial para o desenvolvimento sustentável e a recuperação da pandemia de COVID-19 ^9^. No ano seguinte, a OPAS adotou a Estratégia para a Melhoria da Saúde Mental e Prevenção do Suicídio na Região das Américas ^10^, com o objetivo de “*melhorar a saúde mental e a prevenção do suicídio, utilizando uma abordagem baseada na equidade e nos direitos humanos, a fim de promover a saúde e o desenvolvimento na Região*”. As recomendações finais da Comissão de Alto Nível da OPAS sobre Saúde Mental e COVID-19, delineadas no relatório *Uma Nova Agenda para a Saúde Mental nas Américas*, propulsionaram essa Estratégia ^11^.

Mais recentemente, o impulso regional para a saúde mental atingiu níveis sem precedentes por meio da inclusão do tema na agenda hemisférica da Organização dos Estados Americanos (OEA). Em junho de 2025, os Estados-Membros da OEA aprovaram por unanimidade uma resolução histórica ^12^ para enfrentar a crise de saúde mental nas Américas, que estabelece uma parceria regional para fortalecer a colaboração, expandir o acesso e promover a implementação de iniciativas comunitárias de saúde mental. A resolução também determina a formação de um grupo de trabalho interamericano para desenvolver um plano de ação alinhado à estratégia regional de saúde mental, bem como a criação de um fundo regional de saúde mental para reforçar iniciativas prioritárias. A aprovação desta resolução constitui um passo importante ao colocar a saúde mental na agenda política mais ampla das Américas, onde a saúde mental tem sido historicamente considerada uma questão exclusivamente do setor da saúde.

Embora o comprometimento político em nível regional tenha crescido, é mais difícil mensurar como esse progresso se traduz em nível nacional. Os países da região certamente realizaram avanços cruciais no fortalecimento de seus sistemas de saúde mental nos últimos anos, especialmente por meio do desenvolvimento e atualização de legislação e políticas de saúde mental mais alinhadas aos padrões internacionais de direitos humanos e na transição para abordagens comunitárias de assistência à saúde mental. Um pilar central desse trabalho tem sido a integração dos serviços de saúde mental à atenção primária, utilizando o Programa de Ação para a Lacuna em Saúde Mental da OMS ^13^. É importante ressaltar que quase todos os países da região já realizaram capacitações relacionadas ao programa. No entanto, como apontam Queiroz et al. ^2^, existem inúmeros desafios para a implementação desse programa em diferentes contextos e poucos estudos avaliaram seu impacto em termos do maior acesso aos serviços de saúde mental na assistência primária.

De fato, todos os três estudos citados acima apontam para a necessidade de ampliar as pesquisas em saúde mental para fornecer informações que possam servir de base para políticas eficazes direcionadas a onde a necessidade é maior. A ciência da implementação, em particular, é essencial para avaliar, adaptar e ampliar práticas e ferramentas baseadas em evidências para a saúde mental em diversos contextos locais da região. Maiores investimentos em saúde mental - tanto financeiro quanto na forma de desenvolvimento da força de trabalho - além da colaboração entre setores, incluindo, entre outras coisas, a resposta preparada a emergências, a prevenção da violência e a garantia da justiça, são necessários para reduzir a grande lacuna no tratamento de saúde mental nas Américas e garantir que ninguém seja deixado para trás.

A Região das Américas encontra-se em momento crucial em seus esforços para transformar a saúde mental em uma prioridade da saúde pública e do desenvolvimento. Embora a carga dos distúrbios mentais continue a aumentar, os últimos anos testemunharam um impulso político para a saúde mental. Olhando para o futuro, o desafio torna-se como traduzir esse compromisso em mudanças efetivas em nível nacional, uma tarefa que exigirá vontade política sustentada, investimento e ação multissetorial, bem como dados, evidências e pesquisas para orientá-la.

# Translation: Promoviendo la salud mental en las Américas: retos y oportunidades

**DOI:** 10.1590/0102-311XES162325

**Authors:** Alessandra Trianni, Amy Tausch, Renato Oliveira e Souza

**Affiliations:** 1 Pan American Health Organization, Washington DC, U.S.A.

La salud mental se ha convertido en una prioridad urgente en la Región de las Américas, en parte debido al reconocimiento de los efectos generalizados de la pandemia de COVID-19 en la salud mental y el bienestar de las personas. Sin embargo, a pesar de la importante discapacidad y mortalidad prematura asociadas a los trastornos de salud mental, los países de la Región solo destinan una pequeña parte de sus presupuestos de salud a la salud mental, y la mayoría de la población tiene un acceso limitado a los servicios comunitarios de salud mental. Los datos, aunque escasos, indican que las poblaciones que viven en condiciones de vulnerabilidad se enfrentan a mayores obstáculos para acceder a estos servicios. Serpeloni et al. ^1^, Queiroz et al. ^2^ y Orth et al. ^3^ destacan las deficiencias en la atención de la salud mental de las poblaciones en condiciones vulnerables, en particular las afectadas por conflictos y violencia, y las personas encarceladas, subrayando la necesidad de reforzar la inversión, la colaboración intersectorial y la investigación en materia de salud mental con el fin de ampliar los servicios y llegar a quienes más los necesitan. Los recientes avances en materia de políticas a nivel regional representan una importante oportunidad para hacerlo.

La carga de enfermedad atribuible a los trastornos de salud mental en las Américas es elevada y sigue aumentando. Los trastornos mentales, neurológicos y por consumo de sustancias (MNS) y el suicidio representan más de un tercio de los años vividos con discapacidad (AVD) y casi una quinta parte de todos los años de vida ajustados por discapacidad (AVAD) en la Región ^4^. Entre 2000 y 2019, la tasa de mortalidad por trastornos MNS aumentó en un 89% y la tasa de AVAD en un 10% ^5^. La salud mental también es un factor de riesgo para el suicidio, que solo en 2021 costó la vida a 100.000 personas en la Región. Resulta preocupante que en América se haya registrado un aumento del 17% en la tasa regional de suicidios entre 2000 y 2021, siendo esta la única región de la Organización Mundial de la Salud (OMS) que experimentó un aumento durante este período ^6^. La pandemia de COVID-19 agravó aún más la situación de salud mental en la Región al amplificar los factores de riesgo conocidos para los trastornos de salud mental, como el aislamiento social, el desempleo, la pobreza y la violencia, y al perturbar los ya frágiles sistemas y servicios de atención de salud mental. En 2020, se estimó que los trastornos depresivos mayores y los trastornos de ansiedad aumentaron en un 35% y un 32%, respectivamente, en América Latina y el Caribe debido a la pandemia ^7^.

A pesar de esta carga, sin embargo, los sistemas de salud mental en la Región siguen con financiación crónicamente escasa; solo se destina una media del 3% de los presupuestos nacionales de salud a la salud mental, de la cual casi la mitad se destina a instituciones psiquiátricas de larga estancia, a menudo asociadas con malos resultados y violaciones de los derechos humanos ^8^. En muchos países, la disponibilidad y accesibilidad de los servicios de salud mental comunitarios son muy limitadas, lo que se refleja en una elevada brecha en el tratamiento (se estima que solo el 18% de las personas que padecen psicosis reciben tratamiento) ^8^. Es probable que la brecha sea mayor para determinados grupos de población, pero la falta de datos desglosados dificulta su cálculo.

Reconociendo la creciente crisis de salud mental en la Región, en 2023, los Estados Miembros de la Organización Panamericana de la Salud (OPS) adoptaron la Política para Mejorar la Salud Mental, que enmarcó explícitamente la salud mental como esencial para el desarrollo sostenible y la recuperación de la pandemia de COVID-19 ^9^. Al año siguiente, adoptaron la Estrategia para Mejorar la Salud Mental y la Prevención del Suicidio en la Región de las Américas ^10^, con el objetivo de “*mejorar la salud mental y la prevención del suicidio, utilizando un enfoque basado en la equidad y en los derechos humanos, con el fin de promover la salud y el desarrollo en la Región*”. Un factor clave detrás de la Estrategia fueron las recomendaciones finales de la Comisión de Alto Nivel de la OPS sobre Salud Mental y COVID-19, descritas en el informe de la Comisión, una nueva agenda para la salud mental en las Américas ^11^.

Más recientemente, el impulso regional en materia de salud mental alcanzó niveles sin precedentes al incluirse en la agenda hemisférica de la Organización de los Estados Americanos (OEA). En junio de 2025, los Estados miembros de la OEA aprobaron por unanimidad una resolución histórica ^12^ para abordar la crisis histórica de salud mental en las Américas. La resolución establece una alianza regional en materia de salud mental para fortalecer la colaboración, ampliar el acceso y promover la implementación de iniciativas de salud mental basadas en la comunidad de la región. También exige la formación de un grupo de trabajo interamericano para desarrollar un plan de acción regional alineado con la Estrategia regional de salud mental, así como la creación de un fondo regional de salud mental para reforzar las iniciativas prioritarias en materia de salud mental. Su aprobación supone un paso notable para situar la salud mental en la agenda política general de las Américas, donde históricamente se ha considerado una cuestión exclusiva del sector sanitario.

Si bien el compromiso político a nivel regional ha crecido, es más difícil medir cómo se traduce este progreso a nivel nacional. Sin duda, los países de la región han logrado avances cruciales en el fortalecimiento de sus sistemas de salud mental en los últimos años, especialmente mediante la elaboración y actualización de leyes y políticas de salud mental que se ajustan mejor a las normas internacionales de derechos humanos, y en la transición hacia enfoques comunitarios de la atención a la salud mental. Un pilar fundamental de este trabajo ha sido la integración de los servicios de salud mental en la atención primaria mediante el Programa de Acción Mundial para Superar las Brechas en Salud Mental (mhGAP, por su sigla en inglés) de la OMS ^13^. Es importante destacar que, hasta la fecha, casi todos los países de la Región han llevado a cabo actividades de fortalecimiento de la capacidad en el marco del mhGAP. Sin embargo, como señalan Queiroz et al. ^2^, existen numerosos retos para su implementación en diferentes entornos y pocos estudios que evalúen su impacto en términos de mejora del acceso a los servicios de salud mental en el ámbito de la atención primaria.

De hecho, los tres estudios apuntan a la necesidad de mejorar la investigación en el campo de la salud mental para fundamentar políticas eficaces que se centren en las áreas donde la necesidad es mayor. La ciencia de la implementación, en particular, es esencial para evaluar, adaptar y ampliar las prácticas y herramientas basadas en la evidencia para la salud mental en diversos entornos locales de toda la Región. Es necesario aumentar la inversión en salud mental, tanto financiera como en forma de desarrollo de la fuerza laboral, además de la colaboración entre sectores, incluyendo, entre otros, la preparación y respuesta ante emergencias, la prevención de la violencia y la justicia, para reducir la gran carencia de tratamiento de salud mental en las Américas y garantizar que nadie se quede atrás.

La Región de las Américas se encuentra en una encrucijada decisiva en sus esfuerzos por convertir la salud mental en una prioridad de salud pública y desarrollo. Si bien la carga de las enfermedades mentales sigue aumentando, en los últimos años se ha observado un impulso político sin precedentes en favor de la salud mental. De cara al futuro, el reto sigue siendo traducir este compromiso en un cambio efectivo a nivel nacional, una tarea que requerirá una voluntad política sostenida, inversiones y acciones multisectoriales, así como mejores datos, pruebas e investigaciones que sirvan de orientación.
